# Recent advances in essential oils and their nanoformulations for poultry feed

**DOI:** 10.1186/s40104-024-01067-8

**Published:** 2024-08-10

**Authors:** Fatemeh Movahedi, Nilesh Nirmal, Pengyuan Wang, Hongping Jin, Lisbeth Grøndahl, Li Li

**Affiliations:** 1 Australian Institute for Bioengineering and Nanotechnology, The University of Queensland, Brisbane, QLD 4072 Australia; 2 Institute of Nutrition, Mahidol University, 999 Phutthamonthon 4 Road, Salaya, Nakhon Pathom, 73170 Thailand; 3 Oujiang Laboratory; Key Laboratory of Alzheimer’s Disease of Zhejiang Province, Institute of Aging, Wenzhou Medical University, Wenzhou, 325000 Zhejiang China; 4 JECHO Biopharmaceuticals Co., Ltd., No. 2633, Zhongbin Avenue, Sino-Singapore Tianjin Eco-city, Binhai New Area, Tianjin, China; 5 School of Chemistry and Molecular Biosciences, The University of Queensland, Brisbane, QLD 4072 Australia

**Keywords:** Antibacterial activities, Antioxidants, Essential oils, Nanoformulations, Poultry additives

## Abstract

Antibiotics in poultry feed to boost growth performance are becoming increasingly contentious due to concerns over antimicrobial resistance development. Essential oils (EOs), as natural, plant-derived compounds, have demonstrated antimicrobial and antioxidant properties. EOs may potentially improve poultry health and growth performance when included in poultry feed. Nevertheless, the incorporation of EOs as nutritional additives is hindered by their high volatility, low water solubility, poor intestinal absorption, and sensitivity to environmental conditions. Recently, nanoencapsulation strategies using nanoformulations have emerged as a potential solution to these challenges, improving the stability and bioavailability of EOs, and enabling targeted delivery in poultry feed. This review provides an overview of the antioxidant and antibacterial properties of EOs, the current limitations of their applications in poultry feed, and the recent advancements in nano-engineering to overcome these limitations. Furthermore, we outline the potential future research direction on EO nanoformulations, emphasizing their promising role in advancing sustainable poultry nutrition.

Highlights

• Essential oils (EOs) are known as powerful antioxidants and antibacterial agents.

• EOs have a high potential to replace antibiotics as feed additives.

• Nanoformulations of EOs have shown improved bioactivity and storage stability of EOs.

• Nanoformulation promotes the bioavailability and gut adsorption of EOs as feed additives.

## Introduction

Poultry is the most consumed livestock and a swiftly expanding meat industry globally. To prevent pathogens, boost health, and lower mortality rates in poultry, conventional antibiotics have been extensively employed [1]. Moreover, antibiotics aid in growth, hatchability, productivity, and feed-to-meat conversion [2]. Nonetheless, pervasive antibiotic use has led to the emergence of antimicrobial resistance (AMR)—a significant worldwide concern [3]. AMR in poultry pathogens not only results in treatment inefficacy and financial losses but also facilitates the transmission of resistant genes or bacteria to humans [4]. Consequently, in-feed antibiotics are prohibited in the European Union, Australia, and other countries. For example, European Union has prohibited the routine feeding of antibiotics to farmed animals since 2022.

Restrictions to the use of conventional antibiotics have stimulated the exploration of alternative additives. In this regard, plants and their derivatives, specifically essential oils (EOs), have gained much interest due to their antimicrobial and antioxidant activities, and immunity-boosting and growth-promoting properties [5]. EOs are oily liquids certified as Generally Recognized As Safe (GRAS) by the Food and Drug Administration (FDA) [6]. Thus, EOs can be considered as potential poultry food supplements. However, their low solubility and storage instability hinder their usage. To address this, material scientists have developed nanoformulations for EOs, including nanoemulsions, liposomes, and solid lipid nanoparticles (SLNs), to enhance their bioavailability and stability [7]. Furthermore, these nanoformulations serve as protective barriers, shielding EOs from degradation and environmental factors. They prolong the shelf life of EOs and ensure gradual release within poultry’s digestive tract, intensifying the effects of EOs. Therefore, the EO nanoformulations can be considered as a strategy to promote healthier flocks, safer food production, and sustainable agriculture.

With the rapid growth of the use of EOs in poultry feed and research into nanoformulations of EOs, this review will summarize the recent progress of the applications and limitations of EOs in animal feed. Furthermore, it will provide an extensive overview of developments in EO nanoformulation over the past 5 years, along with their practical application in poultry feed.

## Features and biological activities of EOs

**Fig. 1:**
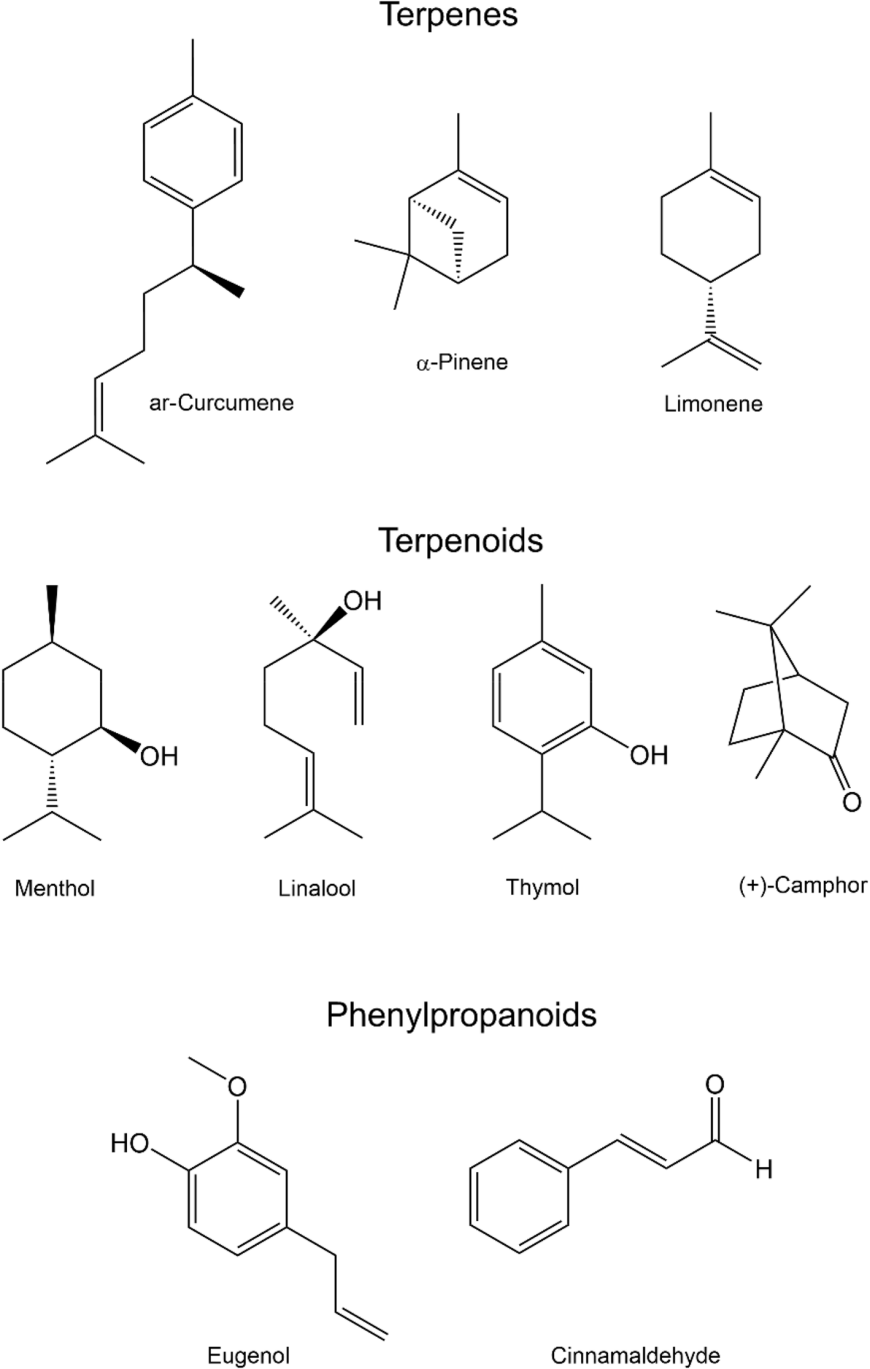
Chemical structures of selected natural products of EOs

EOs, complex mixtures of volatile compounds, are mainly secreted by glandular trichomes, which are the secretory tissues diffused into the surface of plant organs such as leaves, flowers, roots, fruits, or stems [8]. The natural products of EOs have a diverse range of chemical structures, where the majority are categorized into terpenes, terpenoids, and phenylpropanoids, as illustrated in Fig. 1. Terpenes are hydrocarbons that contain isoprene (C_5_H_8_) units such as α-pinene, limonene, and ar-curcumene [9]. Terpenes are the main compounds of plant oils and are responsible for their distinct aroma and flavor [9]. Terpenoids, also known as isoprenoids, are chemically modified terpenes that have undergone functionalization, such as oxidation or rearrangement of their carbon skeletons [9]. Some examples of terpenoids are menthol, linalool, thymol, and (+)-camphor. Phenylpropanoids are a diverse group of secondary plant metabolites derived from the amino acid phenylalanine. They play vital roles in plant physiology, including defence against herbivores and pathogens, protection from UV radiation, and signalling between plants and their environment [10]. Eugenol and cinnamaldehyde are known examples.

The natural products of EOs have been explored as an alternative to conventional antibiotics and growth promoters and attracted much attention in animal nutrition, including poultry feed, due to their superior anti-inflammatory, antimicrobial, and antioxidant properties [11, 12]. Their biological properties also stimulate gut immunity, promoting overall health and productivity in chickens. The detailed structure and properties of the natural products of EOs have been reviewed in Hyldgaard’s publication [13]. Thus, this review article mainly focuses on their biological properties related to poultry feed.

### Antibacterial activity

Among the various natural products found in EOs, terpenes do not have efficient antibacterial activities compared to conventional antibiotics when applied as single compounds [13]. However, they have shown a synergistic or additive effect of antibacterial activity in combination with other naturally occurring antibacterial compounds, resulting in improved efficacy. Terpenoids and phenylpropanoids exhibit antibacterial effects due to the presence of functional groups, such as hydroxyl groups attached to the phenolic rings [13, 14].

The antibacterial properties of natural products in EOs vary depending on their chemical structure, concentration, and target organism. Numerous studies have assessed the antibacterial activity of EOs, demonstrating oils derived from thyme and eucalyptus as highly potent. These EOs contain potent natural products such as eucalyptol [15], thymol [16], eugenol and carvacrol [17] as listed in Table 1. These compounds have shown antibacterial activity against various pathogens in the poultry industry, including Gram-positive and Gram-negative bacteria. The efficacy of EOs against antibiotic-resistant pathogens has also been confirmed [18, 19].

**Table 1:** Antimicrobial activity of EOs listing plant from which EO is derived and the main active compounds (natural products) of the EOs

Common name of plant	Key natural product(s)	Microorganism	Reference
Camphor laurel	Linalool, eucalyptol	MRSA, *Staphylococcus aureus*, *Enterococcus faecalis*, *Bacillus subtilis*, *Salmonella gallinarum* and *Escherichia coli*	[20]
Citron	Limonene, γ-terpinene	*Escherichia coli*, *Staphylococcus aureus*, *Bacillus subtilis* and *Micrococcus luteus*	[21]
Citrus (including lemon, orange, and bergamot)	Citral, limonene, linalool	*Campylobacter jejuni*, *Escherichia coli* O157, *Listeria monocytogenes*, *Bacillus cereus* and *Staphylococcus aureus*	[22]
Coriander	Linalool	*Bacillus cereus* and *Enterococcus faecalis*	[23]
Eucalyptus	Eucalyptol	*Escherichia coli, Staphylococcus aureus,* and *Pseudomonas aeru-ginosa*	[15]
Lavender	Linalool	Meticillin-resistant *Staphylococcus aureus* (MRSA)	[24]
Lemongrass	Citral	*Candida albicans*	[25]
Mastic (resin)	Myrcene, pinene, limonene	*Clostridium botulinum*	[26]
Oregano	Carvacrol	Meticillin-resistant *Staphylococcus aureus* (MRSA)	[27]
Peppermint	Menthol, menthone	*Staphylococcus aureus*	[28]
Rose	Citrenellol, geraniol	*Escherichia coli*, *Pseudomonas aeruginosa*, *Bacillus subtilis*, *Staphylococcus aureus*, *Chromobacterium violaceum* and *Erwinia carotovora*	[29]
Thyme	Thymol, linalool, carvacrol	*Clostridium perfringens*	[30, 31]
Thyme	Thymol	*Cryptosporidium parvum*	[32]
True cinnamon	Cinnamaldehyde	*Aspergillus niger*, *Aureobasidium pullulans*, *Chaetomium globosum*, *Cladosporium cladosporioides*, *Alternaria alternata*, *Penicillium citrinum*	[33]

The majority of natural products in EOs are hydrophobic, making them more likely to penetrate and disrupt the cell membranes of bacteria [34]. Membrane disruption leads to loss of pH gradient, the collapse of protein pumps, drainage of the adenosine triphosphate (ATP) pool, loss of cellular components, an influx of substances, denaturation, and finally, cell lysis [35, 36]. This mechanism agrees with the higher tolerance of Gram-negative bacteria to most EOs.

### Impact of EOs on non-pathogen microbiota in the gastrointestinal tract (GIT)

The GIT of poultry, which continuously interacts with the microbial population, plays a major role in the host’s health and growth [37]. Considering the antimicrobial properties of EOs, it is critical to ensure that they do not inhibit non-pathogenic bacteria in the GIT. Reports indicate that the growth of non-pathogenic gut bacteria can be improved while pathogens can be suppressed simultaneously. For instance, it is well documented that EOs can inhibit the growth of *Campylobacter*, a major foodborne pathogen while improving the growth of beneficial bacteria through the impact on the intestinal mucosal layer [38]. Eleutherin extract inhibited the growth of *Escherichia coli* and *Staphylococcus* and simultaneously promoted the growth of the non-pathogen bacteria *Lactobacillus acidophilus* [39]. Thymol and carvacrol also modified gut health and improved intestinal structure by reducing pathogens and enhancing the population of non-pathogen bacteria [40]. Though the impact of EOs on non-pathogen microbiota research is limited to a few reports, some EOs have shown great potential in improving the GIT non-pathogen population.

### Antioxidant activity

An interesting feature of EOs is their antioxidant activity. Though the chemical structures of the natural products in EOs are diverse, many of them share common features, such as aromatic rings including phenols as shown in Fig. 1. The antioxidant activity of EOs is attributed to the radical scavenging ability of hydroxyl groups that are part of a conjugated system, such as a phenol that can donate a hydrogen atom with the resulting radical resonance stabilised. Other molecules such as ascorbic acid act by donation of an electron to the free radical with the ultimate oxidation of ascorbic acid to dehydroascorbate [41].

The phenolic compounds of EOs, such as eugenol, thymol, and carvacrol, are found to be the most potent antioxidants [42]. The combination of carvacrol, cinnamaldehyde and capsicum oleoresin resulted in an increased concentration of hepatic antioxidants, including coenzyme Q10, total vitamin E, and carotene in broiler chicken [43]. The antioxidant compounds in poultry meat can be absorbed in the intestine and perform an antioxidant function in human [35].

In addition to antioxidant activity, an anti-inflammatory effect is also reported for EO [44], attributing to their radical scavenging properties [42]. Since the key inflammatory responses arise from the oxidative burst in cells and the formation of free radicals, the antioxidant feature of natural products in EOs can play a pivotal role in neutralizing those compounds and suppressing their destructive effect on cells [45], which can have a positive effect on feed efficiency in poultry [42].

## Impact of EOs on poultry performance and overall health

### The effect of EOs as additives on poultry performance

**Fig. 2:**
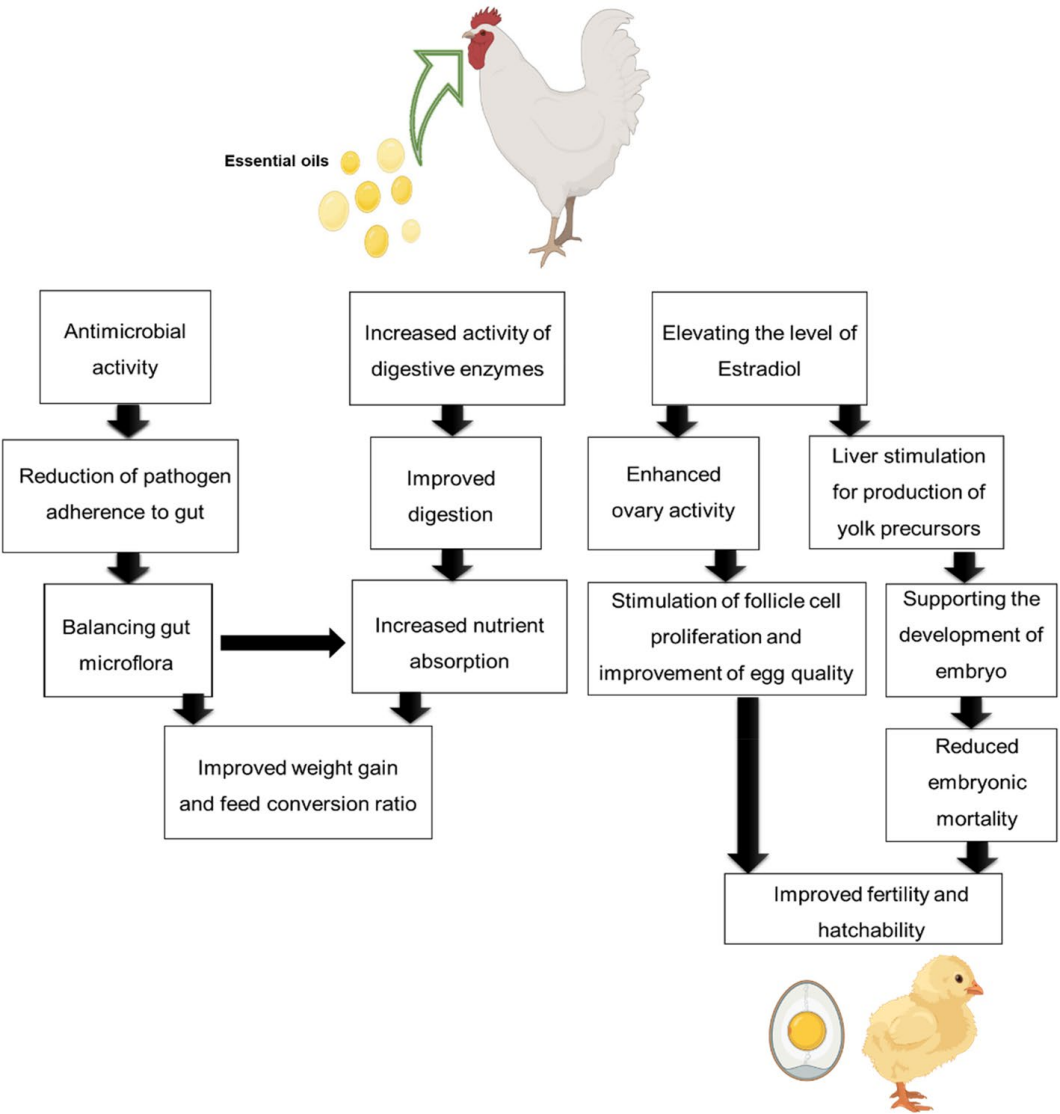
Effect of EOs on poultry performance, including weight gain, feed conversion ratio, fertility, and hatchability. Figure created with BioRender (https://biorender.com/)

EOs are perceived as growth promoters for poultry since they improve weight gain, feed conversion ratio, and the production efficiency index. Enhanced poultry performance is achieved through various mechanisms involving EOs as depicted in Fig. 2. One of the main mechanisms is linked to the antimicrobial activities of EOs. EOs reduce pathogen adherence and balance the gut microflora leading to increased nutrient absorption [46]. A second mechanism relates to stimulating the secretion of digestive enzymes, including amylase and trypsin, as well as hydrochloric acid, resulting in better digestion and nutrient absorption [35, 38]. Specifically, it has been reported that the inclusion of garlic powder or thymol and carvacrol significantly improved the broiler performance and carcass yield [47].

Improvement in fertility and hatchability is reported for broiler chickens whose diet was supplemented with a mixture of active compounds of six EOs, including oregano oil, laurel leaf oil, sage leaf oil, myrtle leaf oil, fennel seed oil, and citrus peel oil [48]. Improved hatchability is similarly reported for quails with thymol and isoeugenol feed supplementation [49]. As illustrated in Fig. 2, increased fertility and hatchability can be attributed to estradiol level elevation. Estradiol stimulates yolk precursor production and improves the activity of the ovary. As a result, embryo development is supported through the stimulation of follicle cell proliferation, ultimately resulting in improved egg quality and quantity [50].

### Immunity boosting

Many researchers have stated the positive effect of EOs on the avian immune system by promoting immunoglobin production and improving lymphocytic activity and interferon secretion [35, 51]. A mixture of carvacrol, thymol, oregano oil, thyme oil, eucalyptus oil, and eucalyptol was assessed in broiler chicken, revealing an immune-stimulative response to the Newcastle Disease (ND) vaccine and an antiviral effect against the ND virus [52]. Peppermint and eucalyptus oils similarly stimulated an innate cell-mediated immune response and humoral immune response in chickens [53]. A combination of black pepper and radish seed oils stimulated the expression of autophagy-related genes and enhanced phagocytosis [54]. Similarly, ginger and thyme oils significantly increased phagocytic activity and antibody titers against infectious bursal disease (IBD) and ND virus in broiler chicken [55].

## Challenges of EOs as additives in poultry feed and desirable nanoformulation solutions

### Challenges of EOs as additives

**Fig. 3:**
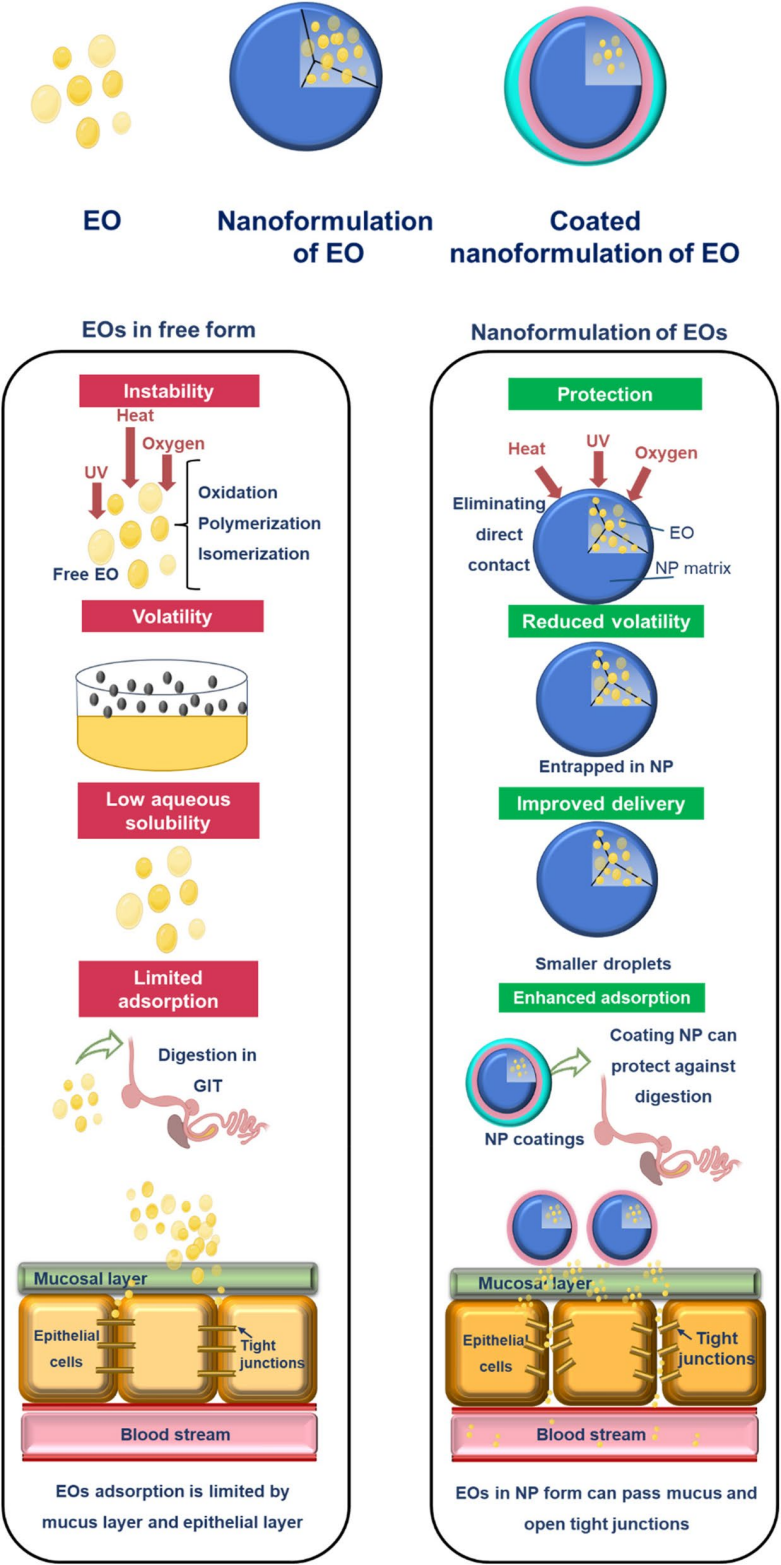
Limitations of EOs in free form and the way nanoformulation can overcome those limitations

Despite the promising attributes of EOs as poultry food additives, their application encounters a range of substantial challenges as presented in Fig. 3. The inherent hydrophobic nature of EOs results in their poor aqueous solubility which significantly limits their dispersibility and homogeneity in feed formulations, potentially leading to inconsistent intake and efficacy in chickens. Moreover, the volatile character of EOs adds another layer of complexity, reducing their stability both during storage and when introduced to the feed. Exposure to environmental factors such as heat, light, and oxygen can trigger their degradation, leading to diminished potency and reduced overall effectiveness [56, 57].

In addition, EOs display limited absorption within the avian gut. This is attributed to their susceptibility to degradation within the complex gut environment, their interaction with mucosal clearance mechanisms, and their slow penetration through the hydrophobic barriers of the gut lining [58]. The challenge of maintaining bioactivity and stability through the intricate journey from production to poultry consumption and adsorption with the gut underscores the need for innovative strategies.

### Current nanoformulations for EOs

Nanoformulations of EOs significantly enhance the effectiveness and stability of these natural products beyond what is achievable with simple EO formulations. Through innovative nanoencapsulation technologies, these formulations are crafted to provide many benefits that address critical challenges in applications like poultry feed, where maintaining the integrity and efficacy of EOs is key. Nanoformulations, possessing a high surface area, offer a higher loading capacity of EOs than their bulk formulations [59]. They may provide a protective shield to EOs, mitigating the adverse effects of environmental factors such as low pH, heat, light, and oxygen that can lead to their deterioration (as illustrated in Fig. 3) [7]. The reduced particle size in nanoformulations enhances the bioavailability of EOs, ensuring that they are absorbed more effectively and interact optimally with microbial membranes in the gastrointestinal tract of poultry, where EOs maintain their antibacterial properties. Additionally, the ability to control the release of EOs at targeted sites allows for prolonged antibacterial and antioxidant effects, ensuring that EOs exert their benefits over extended periods. Within this domain, diverse techniques such as emulsification, nano-precipitation, spray-drying, and inclusion are actively employed to craft nanoformulations of food additives [60]. Coatings such as chitosan not only improve the protection of EOs in the harsh gastrointestinal environment but also enhance their mucosal delivery and intestinal adsorption [58, 60–62].

Another significant benefit of using nanoformulations is the reduced risk of bacterial resistance. EOs in nanoformulations can be taken up by the bacteria through various mechanisms that are distinct from those used by traditional antibiotics, significantly lowering the likelihood of bacteria developing resistance. Nanoparticles can bind to the bacterial membrane through electrostatic interaction, thus disrupting the integrity of the bacterial membrane [63, 64]. Some research works reported that nanotoxicity is typically triggered by the induction of oxidative stress due to the free radical formation of EOs [65]. EOs can alter membrane fluidity, rendering it abnormally permeable and resulting in the leakage of radicals, Ca^2+^ ions, cytochrome C, and proteins, similar to the effects observed in oxidative stress [66]. Bacteria are less capable of adapting to physical damages caused by nanostructured surfaces or overcoming the combined impact of multiple EOs delivered in a controlled manner [67]. By reducing the opportunity for bacteria to adapt and develop resistance, nanoformulations ensure long-term efficacy in combating microbial infections [68]. Overall, the multifaceted improvements offered by nanoformulations of EOs not only enhance their practical applications but also contribute to sustainable practices in industries like poultry farming by minimizing the reliance on traditional antibiotics and reducing the risk of developing antibiotic-resistant bacterial strains.

**Fig. 4:**
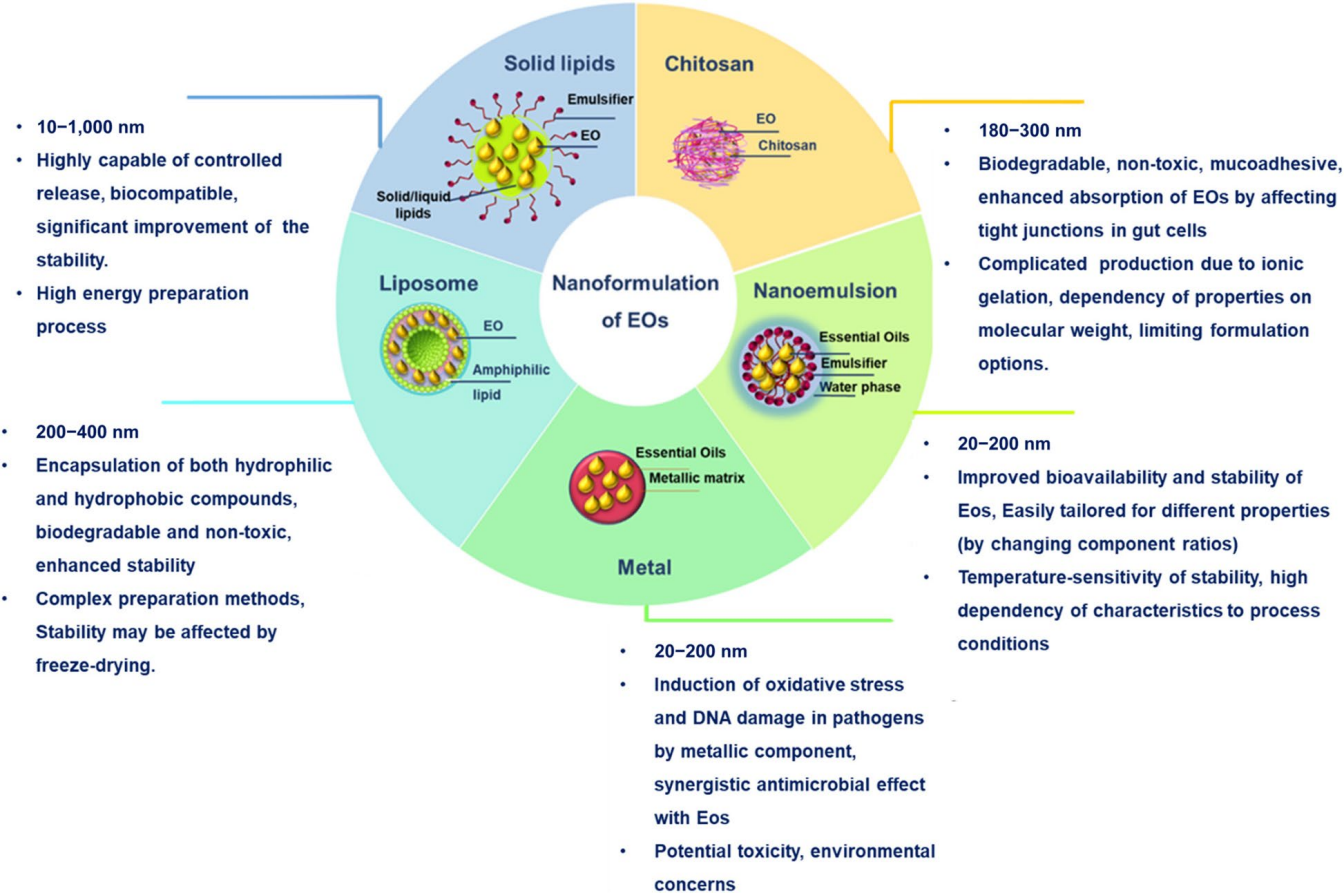
Nanoformulations of EOs for poultry food and their characteristics

Recognizing the critical impact of nanoformulations, Fig. 4 illustrates a variety of EO nanoformulations utilized in poultry feed, each with distinct characteristics. The array includes chitosan NPs, nanoemulsions, liposomes, solid lipid NPs and nanostructured lipid carriers, as well as metal and metal oxide NPs. The following section will delve into the classifications, attributes, and operational functions of these NPs, emphasizing their capacity to advance poultry dietary regimes and health oversight.

#### Chitosan NPs

Chitosan is a polysaccharide composed of glucosamine and *N*-acetyl-D-glucosamine units, approved by the FDA as a food ingredient [69]. Due to its affordability, biodegradability, non-toxicity, cationic nature, and mucoadhesive properties, as well as antifungal and antimicrobial characteristics and capacity to encapsulate hydrophobic molecules, chitosan in NP formulation has been used for the delivery of EOs [70–72].

Chitosan NPs are generally synthesized by ionic gelation using polyanionic tripolyphosphate (TPP) [73–75]. Various EOs, such as those derived from oregano, thyme, clove and saffron, have been incorporated into chitosan NPs, and some have been assessed in the poultry industry [69, 75, 76]. Compared to free EOs, chitosan-nanoencapsulated EOs such as thymol oil, oregano oil, and cinnamon oil have demonstrated higher efficacy [69, 73, 76]. For instance, the use of nanoencapsulated cumin oil at a dose of 200 mg/kg resulted in excellent outcomes compared to other treatments, including the antibiotic growth promoter flavophospholipol at 650 mg/kg, 150 mg/kg of chitosan NPs, and free-form cumin oil at doses of 100 or 200 mg/kg [77]. Specifically, the nanoencapsulated cumin oil increased body weight gain, improved feed conversion ratio, and enhanced *mucin 2* gene expression. The average body weight gain in the finisher period was 67.9 and 72.2 g/b/d for 100 and 200 mg/kg free cumin oil compared to 75.7 and 77.07 g/b/d for 100 and 200 mg/kg nanoencapsulated cumin oil. *Mucin 2* gene expression (log10) was increased from 1.84 for 100 and 200 mg/kg free cumin oil to 1.86 and 1.88 for 100 and 200 mg/kg nanoencapsulated cumin oil, respectively. Moreover, total serum triglyceride and low-density lipoprotein cholesterol levels were significantly lower in birds treated with nanoencapsulated cumin oil compared to the control group. The use of nanoencapsulated cumin oil also resulted in a sustained broiler immune response, as evidenced by changes in IgG, heterophilus (H), lymphocyte (L), and H/L ratio. These findings suggest that nanoencapsulated cumin oil could be an alternative to antibiotics [77].

In a separate study, birds that received both free and chitosan nanoencapsulated form of mint, thyme, and cinnamon oil experienced significant improvements in body weight gain and feed conversion ratio. Among all EOs tested, thyme oil showed the most promising results, which were further enhanced by the nano-encapsulation of chitosan NPs. Additionally, the EOs encapsulated chitosan NPs, particularly those derived from thyme and cinnamon, increased serum IgY and IgM levels and higher populations of *Lactobacillus* spp. in the intestine of broilers. These formulations also had the greatest effect on elevating IgY42 concentrations and enhancing healthy microbial populations [78]. Similarly, the encapsulation of garlic oil in chitosan NPs significantly improved antibacterial and antioxidant properties. Specifically, at a 100 mg/kg dose, the nanoencapsulated garlic oil demonstrated notable benefits in enhancing body weight gain and feed conversion ratio. Moreover, the nanoencapsulated garlic oil positively impacted the *Lactobacilli* population in the digesta of ileo-caecum and *mucin 2* gene expression [65].

Chitosan NPs can improve the absorption of EOs by opening tight junctions in gut cells. Rosenthal et al. [79] reported that chitosan nanoformulation can rapidly decrease transepithelial resistance, and this effect was reversible following wash-out. Moreover, the amino groups of chitosan enable it to interact with negatively charged mucin molecules, leading to enhanced retention on the mucosal surface. This interaction, coupled with chitosan’s ability to crosslink with other molecules in the mucus layer, makes it an effective mucoadhesive material [80]. Chitosan with higher molecular weight is reported to have better mucoadhesive properties [7].

#### Nanoemulsions

Nanoemulsion (NE) in the context of EO encapsulation is the colloidal dispersion of hydrophobic EOs in the aqueous phase to form droplets on the nanoscale, which are stabilized by surfactants [81]. Various techniques, such as nanoprecipitation, inclusion complexation, and solvent evaporation-emulsification, can be used to prepare NEs [9, 82]. NE generally contain oil, surfactant, and co-surfactants. The oil and surfactant type, the water-to-oil ratio, and the oil-to-surfactant ratio significantly affect the physiochemical properties and stability of NE and EOs encapsulation. Moreover, it is thought that volatile EOs interact with the surfactant of nanoemulsions or the cavities of NPs, which enhances their stability and retainment in feed [83]. NEs improve bioavailability in the GIT. It furthermore enhances storage stability, as reported for zedoary turmeric oil which was stable at room temperature for 1 year. The amount of six main active compounds of zedoary turmeric oil in NE form did not show any alteration during this time [84].

Besides the enhanced stability and bioavailability of EOs, NE can enhance their efficacy. For instance, lemon and anise myrtle oils were mixed with 10% surfactant (Tween 80 and Span 80) at a concentration of 1% using ultrasonication to produce NEs [85]. The resulting NEs demonstrated good stability at 4 °C, 25 °C, and 40 °C during storage, except for the lemon myrtle nanoemulsion at 40 °C. The lemon myrtle oil-NE exhibited improved antibacterial activity against both Gram-negative and Gram-positive bacteria (*S. aureus*,* L. monocytogenes*,* E. coli*,* P. aeruginosa*) compared to the EO alone. Similarly, NE of cinnamon bark oil, prepared using ultrasonication with Tween 20 and Span 80, was more effective in inhibiting and inactivating microorganisms such as *E. coli*,* S. aureus*, and *S. cerevisiae* than the crude cinnamon bark oil [86]. NEs of peppermint oil decreased the mortality and *Clostridium perfringens* counts in cecal samples and improved the growth performance of broiler chicken [60].

Through ultrasonication, Ibrahim et al. [87] produced thymol-NE formulations using sodium alginate and Tween 80. The impact of these formulations on the growth performance and protection against *Salmonella* Typhimurium infection in the broiler was demonstrated. The boiler groups fed the diet supplemented with thymol NE containing 0.5% or 1% of thymol oil showed enhanced body weight gain with a better feed conversion ratio even after being challenged with *Salmonella* Typhimurium. The upregulation of digestive enzyme genes (*AMY2A*,* PNLIP*, and *CCK*) and expression of *mucin 2*,* FABP2*,* IL-10*,* IgA*, and tight junction protein genes, along with the downregulation of *IL-2* and *IL-6* genes, contributed to enhanced body weight gain. Furthermore, the prevention of *Salmonella* Typhimurium infections was dose-dependent, with the range of 0.25% to 1% of thymol in diet being effective. Along with a decrease in *S.* Typhimurium populations with 1% thymol in diet, 0.5% and 1% thymol NE in diet led to an increase in *Lactobacilli counts*. The researchers also found that the expression of the *invA* gene was downregulated post-infection [87].

#### Liposomes

Liposomes are non-toxic, safe, biodegradable NPs composed of one or more phospholipid layers surrounding an aqueous core [88]. While the aqueous core can encapsulate water-soluble cargo, the lipid layer can entrap hydrophobic compounds such as EOs [89].

Liposomes can be prepared by thin film hydration, freeze and thaw, the rapid expansion of supercritical solution and precipitation from a gas-saturated solution [88]. The size and properties of liposomes can be controlled by adjusting the composition of the phospholipids used in their preparation [90]. For example, liposomes made from phospholipids with longer fatty acid chains tend to be more stable and less prone to leakage, while those made from phospholipids with shorter chains may be more fluid and more easily deformable [91]. The most commonly used phospholipids are phosphatidylcholine, phosphatidylserine, and phosphatidylethanolamine. Surface-modifying agents, such as polyethylene glycol (PEG), can be incorporated into liposomes to improve their circulation time and reduce their clearance by the immune system [92]. In addition to phospholipids, liposomes may contain cholesterol, which stabilize the lipid bilayer and regulate its fluidity [93]. Optimization of liposomal formulations of eugenol, thymol, guaiacol, trans-anethole and eugenyl acetate with regard to phospholipid to cholesterol ratios, found that a formulation containing 40% cholesterol yielded the most stable liposomes.

Lipid S100-liposomes produced using the ethanol injection method efficiently encapsulated estragole, eucalyptol, isoeugenol, pulegone, terpineol, and thymol [94]. The drug release rate from the liposomes was controlled by the loading rate of the natural products into the liposomes and their location within the lipid bilayer, the cholesterol incorporation rate and the size of the liposomes. Furthermore, a considerable amount (> 50%) of isoeugenol, pulegone, terpineol, and thymol were preserved in the liposomes for up to 10 months, indicating the long-term stability of the liposomal formulation.

To enhance the stability of clove oil and its primary component, eugenol, natural soybean phospholipid vesicles were developed using the ethanol injection method. Liposomes were prepared by combining saturated (Phospholipon 80H and 90H) and unsaturated soybean (Lipoid S100) phospholipids with cholesterol at varying concentrations of eugenol and clove oil [95]. The stability of the liposomes was evaluated by monitoring changes in their mean size, polydispersity index, and encapsulation efficiency values after storing them for 2 months at 4 °C. The liposomes effectively protected eugenol oil from degradation induced by UV exposure and maintained its radical-scavenging activity.

According to Lin et al. [96], some liposomes were exposed to freeze-drying due to their convenience in storage, resulting in enhanced stability of eucalyptus oil compared to those in liquid form. The stability evaluation was performed weekly by monitoring alterations in particle size, polydispersity index (PDI), and zeta potential. It was observed that the inclusion of β-cyclodextrin as a cryoprotectant during freeze-drying was efficacious.

A layer-by-layer deposition method was employed to enhance the stability of chrysanthemum oil liposomes [97]. Liposome coated with chitosan, pectin, and chitosan (triple layer) had an increased particle size of 2.15 µm (PDI of 0.183) compared to the single layer liposome (size 132 nm and PDI of 0.152) as well as a less negative zeta potential of –15 mV compared to –38 mV. The triple-layer liposome exhibited greater stability (2-week storage period at a temperature range of 4–37 °C) than the single-layer and double-layer liposomes and demonstrated high antibacterial activity against *Campylobacter jejuni* in chicken.

Liposomal formulations of EOs have demonstrated improved antimicrobial and antiviral activities against various pathogens. For instance, liposomal formulations of clove oil and cinnamon oil demonstrated improved antimicrobial activity against methicillin-resistant *S. aureus* [98], and liposomal nutmeg oil, and lemongrass oil showed enhanced antimicrobial activity against *Listeria monocytogenes* [99, 100]. Moreover, liposomal *Artemisia arborescent* L. enhanced the antiviral activity against Herpes simplex virus type 1 (HSV-1) [101].

#### Solid lipid NPs and nanostructured lipid carriers

SLNs and nanostructured lipid carriers (NLCs) with a size range of 10–1,000 nm have been developed to encapsulate natural hydrophobic compounds [102]. SLNs and NLCs can be prepared by various techniques, including high-pressure homogenization, solvent emulsification-evaporation, microemulsion-based techniques, supercritical fluid technology and hot homogenization and ultrasonication. EO encapsulation in SLNs or NLCs can be tailored by the choice of lipids and surfactants. For example, lipids with higher melting points may be used for EOs with high volatility to prevent their rapid release from the particles. Similarly, surfactants with higher hydrophilic-lipophilic balance (HLB) values may be used for oils with low water solubility to improve their stability and dispersion in aqueous systems [103].

SLNs have a solid lipid core surrounded by a layer of surfactant molecules and can be formulated with various lipids, such as triglycerides, fatty acids, and waxes [104]. They have shown several benefits, such as biocompatibility, controlled release, and improved stability of the encapsulated drugs [105]. In contrast, NLCs are composed of solid and liquid lipids, with the liquid lipids embedded in the solid lipid matrix to increase the drug loading capacity and improve the stability of the NPs [106].

SLNs and NLCs can protect EOs from degradation during storage, thereby improving their efficacy [107]. A mixture of corn, sesamol, sweet almond, black seed oil, cocoa butter and Tween 80 was used to prepare cinnamon-NLCs [108]. The almond oil-based NLC formulation exhibited an average size of 100–120 nm and an encapsulation efficiency of more than 82%. The stability of cinnamon-NLC was evaluated over 40 d, subjected to different pH conditions and pasteurization treatment, which did not statistically alter the particle size and encapsulation stability.

The encapsulation of EOs in SLNs and NLCs has improved their antimicrobial and antifungal activity. For example, SLNs containing eugenia caryophyllata oil prepared by high-shear homogenization and ultrasound method displayed greater antimicrobial activity than the free oil against *Salmonella typhi*,* Pseudomonas aeruginosa*,* Staphylococcus aureus*, and *Candida albicans.* The eugenia caryophyllata oil-encapsulated SLNs showed a significantly lower minimum inhibitory concentration (MIC) than the free eugenia caryophyllata oil [109].

Cationic NLCs can interact with the membrane of Gram-negative bacteria through electrostatic attraction, while anionic NLCs can interact with the bacterial membrane through hydrogen bonds, in both cases leading to bacterial rupture [107]. Ribeiro et al. [110] investigated the antimicrobial activity of three anionic NLCs encapsulating EOs against the Gram-negative bacterium *Campylobacter jejuni*. The EOs were olibanum, salvia, and candeia oil that were encapsulated in NLCs made with 100 mg/mL ucuuba butter (F6), 100 mg/mL shea butter (F14), or 60 mg/mL shea butter (F19), respectively. The disc diffusion test showed that EO-NLCs exhibited higher growth inhibition zones diameters (ranging from 35 to 43 mm) than their respective pure oils (diameters ranging from 21 to 28 mm), demonstrating these EO-NLC formulations as a potential antimicrobial agent against *Campylobacter jejuni.* F6 showed the highest stability of EO-NLCs among the samples, with no significant changes over 1 year.

#### Metal and metal oxide NPs

Encapsulation of EOs in metallic NPs can enhance their antioxidant and antimicrobial effects, similar to other NPs; however, a synergistic effect is often observed for metallic NP formulations of EOs due to the combination of the unique properties of both EOs and metal NPs. For example, the combination of silver NPs and tea tree oil exhibited synergistic effects against *E. coli* at a fractional inhibitory concentration of 0.48 [111]. Similar synergism is reported for eucalyptus leaf oil which decreased the infection and inhibited the growth of bacteria, including *E. coli* O157:H7, *E. coli*, MRSA, *S. enteric*, and *B. subtilis* [112]*.* The combined use of silver NPs and oregano oil resulted in synergistic or at least additive effects against 17 bacterial strains, including both Gram-positive and Gram-negative bacteria, as well as multidrug-resistant strains. According to time-kill curves, the combined EO-silver NPs reduced their MIC values and shortened the time required for action [113]. Zinc oxide NPs have also shown a synergistic antimicrobial effect with fennel oil against *S. aureus*, *E. coli*, and *A. flavus* [114].

The antibacterial synergism of EOs and metallic NPs can be attributed to various mechanisms involving the catalytic activity of metallic NPs causing oxidative stress, DNA damage, metabolic disruption, reactive oxygen species (ROS) induction, and cell wall damage [114, 115]. EOs contribute to antimicrobial synergism by disrupting bacterial cell walls and cell membranes, leading to leakage of intracellular contents and ultimately cell death. The presence of EOs can also enhance the penetration of metallic NPs into bacterial cells, allowing for increased interaction between the NPs and intracellular targets [7].

### Practical applications and challenges of EO nanoformulations in poultry feed

#### Comparison of EO nanoformulations with common feed additives

Nanoformulations of EOs offer several advantages compared to common feed additives such as probiotics, prebiotics, and antimicrobials, as represented in Table 2. The key advantage is their enhanced effectiveness, stemming from improved bioavailability and targeted delivery. Nanoformulations ensure sustained release of active components, leading to prolonged benefits. Moreover, nanoformulated EOs exhibit minimal interactions with feed matrices and other components, ensuring seamless integration into poultry feed without compromising feed quality or nutrient absorption.

**Table 2:** Comparison of nanoformulations of EOs with common poultry feed additives

Aspect	Nanoformulations of EOs	Probiotics and prebiotics	Antimicrobials
Effectiveness	Enhanced antimicrobial and immunomodulatory effects due to improved bioavailability and targeted delivery	Modulation of gut microbiota and immune function, with efficacy influenced by strain selection and environmental conditions	Immediate antimicrobial action, but potential for antibiotic resistance development and limited long-term gut health benefits
Duration of action	Sustained release of active components for prolonged benefits to poultry gut health	May require continuous supplementation to maintain efficacy, with effects diminishing after cessation of administration	Short-term effectiveness, necessitating repeated administration to combat microbial challenges effectively
Compatibility	Improved compatibility with feed matrices and minimal interactions with other feed components, ensuring seamless integration into poultry feed	May interact with feed components, affecting feed quality or nutrient absorption	May negatively impact feed quality or result in residue accumulation in animal products, leading to food safety concerns
Synergistic effects	Potential synergies with other feed additives like probiotics and prebiotics, enhancing overall efficacy in promoting gut health and performance in poultry	Synergistic effects possible when combined, but compatibility with other additives may vary	Limited potential for synergy with other feed additives, with potential for resistance development in microbial populations

In contrast, while probiotics and prebiotics also contribute to health and immune function modulation, their efficacy may be influenced by strain selection and environmental conditions. Continuous supplementation may be necessary to maintain their effectiveness, with benefits diminishing after cessation of administration. Additionally, antimicrobials offer immediate action against microbial challenges but pose risks such as antibiotic resistance development and potential residue accumulation in animal products, raising food safety concerns.

Finally, nanoformulated EOs have the potential for synergistic effects when combined with other feed additives such as probiotics and prebiotics, thereby enhancing overall efficacy in promoting gut health and poultry performance. This synergy, coupled with sustained release and improved compatibility, highlights the advantages of nanoformulations of EOs as poultry feed additives compared to traditional alternatives.

#### Regulatory challenges and requirement for EO nanoformulations in poultry feed

Despite the advantages of nanoformulated EOs over other poultry feed additives, navigating the regulatory landscape presents several challenges. While various EOs, such as clove, oregano, thyme, nutmeg, basil, mustard, and cinnamon, are classified as GRAS by the FDA [116], incorporating them into NP formulations introduces complexities that require careful consideration for regulatory approval. Nanoformulations of EOs are evaluated individually due to the lack of standardized definitions and guidelines for NPs [117]. This lack of uniformity complicates the regulatory process, as each product must undergo thorough evaluation to determine its safety and efficacy. In addition, nanoformulations intended for export or sale in other countries may encounter additional regulatory hurdles. Each country may have its own regulatory framework for approving and labelling poultry feed additives, adding complexity to the approval process [116].

Characterizing NP-based formulations is particularly challenging due to their complex structures and properties [118]. Current techniques may not fully capture their behaviour in physiological environments, necessitating advancements in characterization methods to ensure accurate assessment. Furthermore, traditional safety data based on bulk materials may not accurately reflect the pharmacodynamic and pharmacokinetic activity of nanoformulations [118]. Such variations result in challenges in establishing safety and efficacy parameters, requiring the development of new assays and testing protocols for accurate toxicity assessment.

Ensuring consistency and stability in the scale-up and manufacturing processes of nanoformulations is critical for maintaining their quality, efficacy, and safety. Rigorous protocols are necessary to address variations and ensure product reliability throughout the manufacturing process. Additionally, nanoformulations may exhibit unusual biodistribution patterns and long-term persistence in specific tissues, necessitating comprehensive testing and safety evaluation to understand their potential side effects [117]. Lastly, the use of nanoformulations for EO delivery deviate from systemic delivery, which poses challenges in defining their pharmacokinetics. Regulatory authorities must assess bioavailability and potential health hazards associated with sustained drug release [118].

To address these challenges effectively, collaboration between regulatory authorities, industry experts, and researchers is essential. This collaboration can lead to the development of standardized guidelines, advancements in characterization techniques, and the establishment of comprehensive safety evaluation protocols for nanoformulations of EOs.

#### Consumer perception and acceptance of poultry feed products with nanoformulated EOs

In addition to considering regulatory challenges, addressing consumer perception and acceptance of poultry products derived from bird fed with diets supplemented by nanoformulated EOs is crucial for market acceptance and the success of these innovative feed additives. Based on the insights from Amato et al. [119], it is evident that consumer preferences lean strongly towards attributes such as the type of farming and "Free-from" claims on labels. These preferences underscore the importance of clean labelling and transparency in communicating product qualities to consumers.

Given the general acceptance of natural additives over synthetic alternatives, nanoformulated EOs in poultry diets have a potentially favourable starting point [46]. These formulations are perceived as natural, which may align well with growing consumer demands for products free from synthetic preservatives and chemicals. However, the novelty of "nano" as a concept in food could provoke hesitancy due to inadequate consumer knowledge, thus necessitating strategic educational efforts. Additionally, offering product demonstrations and trials can provide consumers with firsthand experiences of the quality of these poultry products, effectively altering perceptions and fostering acceptance. Engaging with consumer feedback through surveys and social media will help in understanding and addressing their concerns and questions, leading to more targeted communication efforts. Collaborating with food safety regulators and health experts to ensure stringent safety standards, and gaining public endorsements can significantly boost consumer trust. By integrating these strategies, poultry producers can facilitate a smooth introduction of nanoformulated EO supplements into poultry diets, ensuring consumer understanding and acceptance and setting the stage for successful market integration of these innovative products.

## Conclusion and perspective

EOs possess antimicrobial, antioxidant, and anti-inflammatory properties that can positively impact poultry productivity and overall health. Nanoparticle delivery systems for EOs in animal feed are an emerging technology with potential benefits in chicken health, growth performance, and feed efficiency. However, several challenges still need to be addressed to optimize their application and ensure their safety and efficacy, including:

1. Nanoformulation stability in feed matrix: The feed matrix comprises various components, including proteins, carbohydrates, fats, minerals, and vitamins. Interactions between nanoformulations and these components can lead to changes in particle size, encapsulation status, aggregation, or degradation, ultimately affecting the stability of the nanoformulation. Moreover, the feed production process often involves mechanical forces, such as grinding, mixing, and pelleting, which can cause physical damage to the NPs or lead to aggregation, impacting the stability of the nanoformulation. While storage stability is often tested for NP formulations, there is no work done on testing in-feed stability in the presence of these components or after production processes which would be of importance for the application.
2. Bioavailability enhancement: Achieving high bioavailability of the encapsulated EOs is crucial to maximizing their therapeutic effects. Incorporating EOs into nanoformulations can help overcome the bioavailability issues of EOs in free. However, the size and surface properties of nanoformulations and their interactions with the GIT systems affect the bioavailability of EOs. Developing more effective nanoformulations for EOs is an ongoing challenge and future work may benefit for systematically evaluate the effect of surface properties of the EOs.
3. Safety concerns: The use of nanoformulations in animal feed raises potential concerns regarding their safety, both for the animals and for humans who consume animal products. Rigorous toxicological studies are needed to evaluate the safety profile of NP-encapsulated EOs.
4. Cost-effectiveness: Developing and producing nanoformulation delivery systems can be expensive. To be commercially viable, these systems must demonstrate clear benefits in terms of animal health and productivity, as well as cost savings compared to conventional feed additives. More work on scale-up production of nanoformulations is required to assess this issue.
5. Environmental impact: The long-term environmental impact of using NPs in animal feed is not yet fully understood. It is important to study the potential risks of NP accumulation in the environment and the food chain. It is therefore recommended that the focus is on the development of biodegradable and eco-friendly materials.

To address the above challenges, future studies should focus on developing cost-effective nanoformulations with tailored size and surface properties to control the release of EOs at the target site and enhance uptake by the GIT and optimizing encapsulation methods by exploring different preparation techniques (e.g., emulsion, coacervation, and spray drying) and materials (e.g., polymers and lipids) to maximize encapsulation efficiency and protect EOs from degradation. Comprehensive toxicological studies should be conducted to evaluate the potential risks and long-term effects of EO nanoformulations using appropriate animal models and experimental designs on animals, humans, and the environment. The pH-responsive NPs are good candidates to protect EOs in the stomach and deliver active agents to the appropriate gastrointestinal regions. By encapsulating EOs in pH-responsive NPs, the oils can be protected from degradation in the acidic environment of the stomach, allowing them to reach the lower GIT intact. In addition, designing delivery systems with controlled-release properties can help optimize the bioavailability of EOs. Furthermore, developing cost-effective and scalable production methods will make them a viable alternative to conventional feed additives in the poultry industry. This integrated approach will help unlock the full potential of EO nanoformulations in animal feed, ultimately improving animal health, nutrition, and productivity.

## Data Availability

Not applicable.

## Abbreviations

ATP: Adenosine triphosphate
AMR: Antimicrobial resistance
EO: Essential oil
FDA: Food and Drug Administration
GIT: Gastrointestinal tract
GRAS: Generally Recognized As Safe
HSV-1: Herpes simplex virus type 1
HLB: Hydrophilic-lipophilic balance
IBD: Infectious bursal disease
C_5_H_8_: Isoprene
MIC: Minimum inhibitory concentration
NLC: Nanostructured lipid carrier
NE: Nanoemulsion
NP: Nanoparticle
ND: Newcastle Disease
PDI: Polydispersity index
PEG: Polyethylene glycol
SLN: Solid lipid nanoparticle
TPP: Tripolyphosphate
